# Fast detection of tobacco mosaic virus infected tobacco using laser-induced breakdown spectroscopy

**DOI:** 10.1038/srep44551

**Published:** 2017-03-16

**Authors:** Jiyu Peng, Kunlin Song, Hongyan Zhu, Wenwen Kong, Fei Liu, Tingting Shen, Yong He

**Affiliations:** 1 College of Biosystems Engineering and Food Science, Zhejiang University, Hangzhou, 310058, China; 2 School of Information Engineering, Zhejiang A&F University, Lin’an, Hangzhou, 311300, China

## Abstract

Tobacco mosaic virus (TMV) is one of the most devastating viruses to crops, which can cause severe production loss and affect the quality of products. In this study, we have proposed a novel approach to discriminate TMV-infected tobacco based on laser-induced breakdown spectroscopy (LIBS). Two different kinds of tobacco samples (fresh leaves and dried leaf pellets) were collected for spectral acquisition, and partial least squared discrimination analysis (PLS-DA) was used to establish classification models based on full spectrum and observed emission lines. The influences of moisture content on spectral profile, signal stability and plasma parameters (temperature and electron density) were also analysed. The results revealed that moisture content in fresh tobacco leaves would worsen the stability of analysis, and have a detrimental effect on the classification results. Good classification results were achieved based on the data from both full spectrum and observed emission lines of dried leaves, approaching 97.2% and 88.9% in the prediction set, respectively. In addition, support vector machine (SVM) could improve the classification results and eliminate influences of moisture content. The preliminary results indicate that LIBS coupled with chemometrics could provide a fast, efficient and low-cost approach for TMV-infected disease detection in tobacco leaves.

Tobacco mosaic virus (TMV) was firstly discovered in tobacco, and could infect over 350 different species of plants, including crops of tobacco, tomato, pepper, cucumber, etc. As one of the most stable viruses, TMV can survive outside the plant, and remain in a dormant state to infect growing crops. Once the plant is infected, no chemical cure is effectively available, and usually all the infected crops should be removed. Then those suspect crops should also be further diagnosed. It has been reported that TMV could cause severe production loss and affect the quality of tobacco products^1^.

Currently, enzyme-linked immunosorbent assay (ELISA) and polymerase chain reaction (PCR) are the most commonly used methods to detect TMV in plants^2^. However, the processes of those methods are quite time-consuming, complex, and even challenging since the TMV may distribute unevenly in plant tissues at low level. Therefore, those undetected plants may continue to infect other plants and cause potential loss.

Laser-induced breakdown spectroscopy (LIBS) is a novel atomic emission spectroscopy technique, which has the advantages of fast analysis speed, little sample preparation, and multi-element analysis availability. In the past decades, LIBS combined with chemometrics have been proved as an efficient tool to characterize compounds with similar chemical compositions, such as explosive residues^3^, geochemical materials^4^, and pesticide residues^5^. It is mainly credited to ‘fingerprint’ feature of LIBS and ‘data mining’ capability of chemometrics. In previous reports, LIBS has been used to detect Huanglongbing disease in citrus leaves^6^,^7^. Pereira *et al*.^6^ used LIBS and soft independent modeling of class analogy (SIMCA) to classify the diseased sample within different inoculation times, resulting in the classification accuracy of 82–97%. Sankaran and the co-workers^7^ applied LIBS to detect diseases and nutrient deficiencies in citrus leaves, with the overall classification accuracy of 97.5% for support vector machine (SVM) model. Both of these two studies preferred to use fresh leaves for detection, as the former analysed the midrib of leaves, while the latter scanned four different positions in leaves.

However, the moisture content in samples can greatly affect the spectral features. With the increase of moisture content, most spectral intensities decrease greatly and some emission lines even disappear. Furthermore, high moisture content might worsen the signal stability and lower the signal-to-background rate when analysing coal with LIBS^8^. In this study, we utilized LIBS to detect TMV-infected tobacco with different symptoms and analyse influences of moisture content. Two different kinds of samples including fresh leaves and dried leaf pellets of tobacco (i.e., by pressing the grinded dried leaves) were prepared to investigate the effect of moisture content on LIBS detection, and to establish the TMV disease classification models based on full spectrum and observed emission lines of LIBS.

## Results

### Comparison of fresh and dried tobacco leaf samples

#### Raw spectra analysis

**Figure 1:**
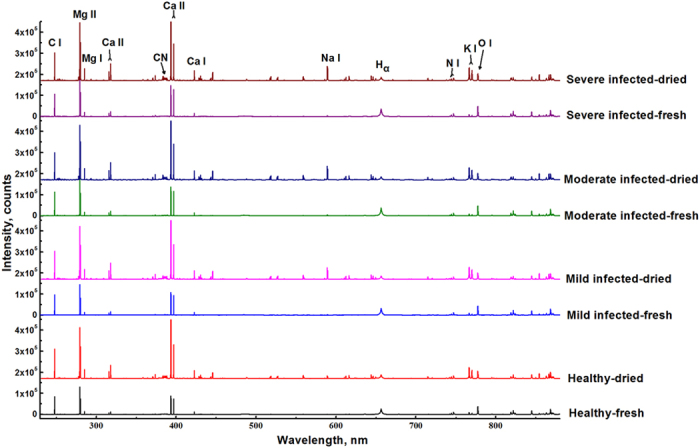
Averaged spectra for healthy and infected tobacco with varying degrees of symptoms. The wavelength region is 230–880 nm. Spectra from both fresh and dried samples showed the similar profile, while the intensities of most emission lines were reduced, and some emission lines were disappeared. There were slight differences in peak intensity and peak position among varying degrees of symptoms.

Figure 1 shows averaged spectra of fresh and dried tobacco leaves with varying degrees of infected symptoms. As seen, both fresh leaves and dried leaves had similar emission lines, which mainly relates to organic compounds and nutritious elements. Molecular bands CN (around 388 nm), which usually associates with organic compounds, were observed in both fresh and dried samples^9^. In addition, some atomic emission lines of C I (247.86 nm), Mg II (279.55 nm, 280.27 nm), Mg I (285.21 nm), Ca II (315. 89 nm, 317.93 nm, 393.37 nm, 396.85 nm), Ca I (422.67 nm), H_α_ (656.28 nm), N I (triplet at 742.36 nm, 744.23 nm, 746.83 nm), K I (766.49 nm, 769.90 nm), O I (unresolved triplet at 777 nm) were easily recognized.

However, some differences were also observed between fresh and dried samples. Table 1 shows main emission lines (the emission intensity higher than 1000 counts) observed in fresh and dried samples. All atomic emission lines were identified using National Institute of Standards and Technology database (http://physics.nist.gov/PhysRefData/ASD/lines_form.html). Some low-intensity emission lines, such as Fe I, Fe II, Mn II, Sc II, Sr II, Ba I, Li I, disappeared in fresh samples. This might be credited to the effect of moisture content in fresh samples. The humility of sample reduced the emission intensities from the plasma^10^. However, the emission intensities of H_α_ (656.28 nm) and O I (unresolved triplet at 777 nm) in fresh leaves were higher than those in dried leaves. It has been reported that there were positive correlations between moisture content and the signal of H_α_ and O I^11^,^12^. These two emission lines might be used to quantify the moisture content.

As for the differences between healthy and infected samples, it was hard to distinguish from peak positions or peak intensities in both fresh and dried samples. Therefore, it was required to use chemometrics to find the differences and achieve a good discrimination.

#### Signal stability analysis

Signal stability is one of important figures of merit in analysis, which usually relates to the repeatability. In this case, the signal stability of some main emission lines was investigated after comparison of raw spectra. Relative standard deviation (RSD) was used to measure the signal stability. All spectra from different positions were used to calculate the value of RSD, and the RSDs of all healthy samples were calculated. Then the values of RSDs were averaged. In order to investigate the stability of raw signal, no preprocessing methods were applied.

**Figure 2:**
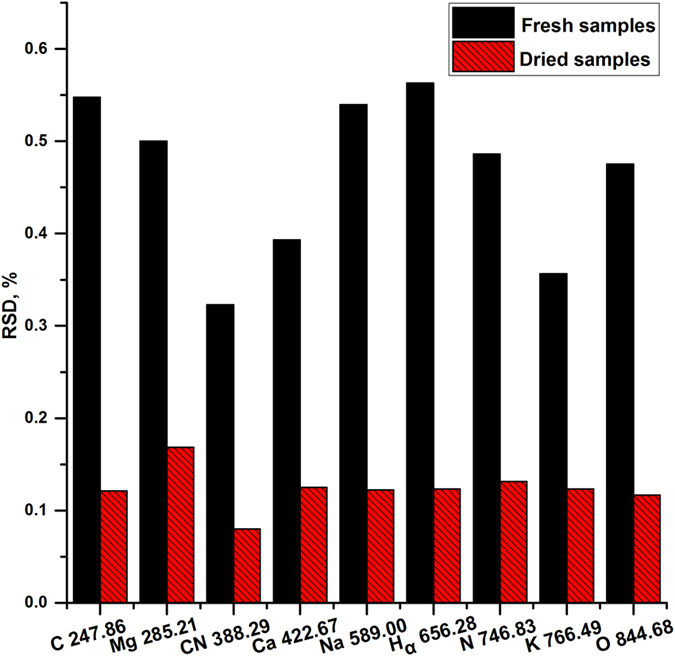
Relative standard deviation of main emission lines from fresh and dried samples (healthy tobacco leaves). Main emission lines from dried samples had lower RSDs than those from fresh samples, which ranged from 5% to 15%.

Figure 2 shows the averaged RSD of main emission lines from healthy tobacco leaves. Obviously, the emission lines from plasma of dried samples had lower RSDs, which ranged from 5% to 15%. The RSDs for fresh samples were relatively high, which ranged from 30% to 60%. There might be two reasons accounting for this. Firstly, dried leaf samples were dried, grinded and pressed, and they had a better micro homogeneity than fresh samples. This might improve the efficiency of ablation, and reduce the matrix effect. In contrast, the moisture content in fresh samples might increase the unevenness of sample surface. Secondly, the moisture content in fresh samples might increase the instability of the plasma, as the evaporation of water generated might splash melted particles^8^.

Among all these main emission lines, CN 388.29 nm had the lowest RSD in both fresh and dried samples. CN emissions appeared in all carbon materials when analysed in the presence of nitrogen. The formation of CN molecular emissions came from two major pathways, by the direct fragmentation of CN bands from the materials, or by recombination of C and N inside the plasma^9^. No matter which pathway it came from, the elements C, N, and molecular band CN band in samples were relatively stable. Therefore, we used CN emission line 388.29 nm as a variable to detect outliers in this experiment.

#### Plasma parameters analysis

In order to investigate the plasma property of fresh and dried samples, temperature and electron density of plasma were calculated. In this study, temperature was obtained by spectrum simulation of the CN emissions (around 388 nm) using LIFBASE 2.1 software^13^. The electron density was determined from Stark broadening of H_α_ with the following equation^14^:


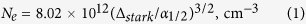


where *N*_e_ is the full width at half maximum (FWHM) of H_α_, α_1/2_ is a weak function of temperature and electron density (in this case, the value was 1.86 × 10^12^ Å)^15^. And the Δ_stark_ is the Stark broadening of H_α_.

Table 2 shows the temperature and electron density of plasma of fresh and dried samples (healthy tobacco leaves). The calculated electron densities in both fresh and dried samples satisfied the McWhirter criterion which suggests the existence of local Thermodynamic Equilibrium (LTE). This condition was crucial for quantitative analysis in LIBS.

In addition, the temperature and electron density in fresh samples were higher than those in dried samples. This was in accordance with previous study, which stated that larger values of temperature and electron density for the samples with higher moisture content were observed at the beginning^8^. Because the fresh tobacco leaves might contain more than 70% moisture content, most of the ablated mass was water. In this case, this evaporated water contributed the higher value of temperature and electron density, and induced stronger emissions of H and O.

### Classification models based on full LIBS spectra

#### PCA analysis

Because it is difficult to distinguish various degrees of symptoms caused by TMA from raw spectra, we used chemometrics to solve this problem. Firstly, principal component analysis (PCA) was applied to qualitatively classify and visualize the distribution of different symptoms in principal component (PC) score plot.

**Figure 3:**
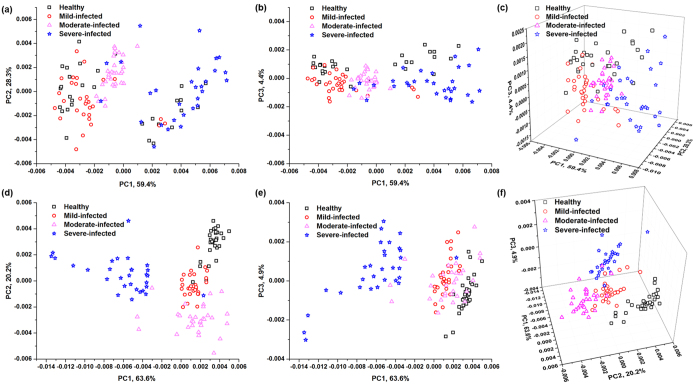
PC score plots for spectral datasets based on fresh samples (**a**) PC1 vs. PC2; (**b**) PC1 vs. PC3; (**c**) PC1, PC2 and PC3) and dried samples (**d**) PC1 vs. PC2; (**e**) PC1 vs. PC3; (**f**) PC1, PC2, and PC3). The first three PCs for fresh and dried samples contributed to 92.1% and 88.7% of the total explained variations, respectively. Better separations could be observed for dried samples.

Figure 3 shows score plots for the spectral datasets based on fresh and dried samples, and each point in the plot represents a sample. Since the first three PCs contained the most of spectral information of tobacco samples, we defined spaces using PC1 and PC2, as well as PC1 and PC3. Compared with fresh samples, dried samples with the same symptom tended to cluster together. As shown in Fig. 2, the emissions from dried samples had lower RSDs than those from fresh samples. It indicated that moisture content might increase the uncertainty of signal, and worsen the performance of clustering. In addition, since the signal from moisture content in fresh samples accounted for the most of plasma, the information representing the features of samples might be reduced.

For dried samples, four clusters were obviously found in the space defined by PC1 and PC2, where healthy samples had distinguished separation with severe-infected samples, while there were slight overlaps between healthy samples and mild-infected samples, mild-infected samples and moderate-infected samples, moderate-infected samples and severe-infected samples. In the score plots of PC1 and PC3, although good separation was found between healthy samples and severe-infected samples, mild-infected samples and moderate-infected samples mixed with each other. Therefore, PC2 might contain more information to separate the mild-infected samples and moderate-infected samples. In addition, the loadings of the first three PCs for both fresh leaves and dried pellets are shown in Supplementary Figure S1. The variables from main emission lines projected to the three PCs, which played an important role in classification.

#### PLS analysis

Partial least squares discrimination analysis (PLS-DA) was further utilized to quantitatively examine the separability of full spectrum using full-cross validation strategy. Before modeling, we split 120-sample dataset into calibration set (84 for dried pellets, and 82 for fresh leaves) and prediction set (36 for dried pellets, and 38 for fresh leaves) using k-Means clustering, with similar proportions of the samples within each degree assigned to each set. The latent variables (LVs) for fresh and dried samples were optimized to ‘7’ and ‘10’, respectively, when the minimal mean squared error of the full-cross validation was obtained.

**Figure 4:**
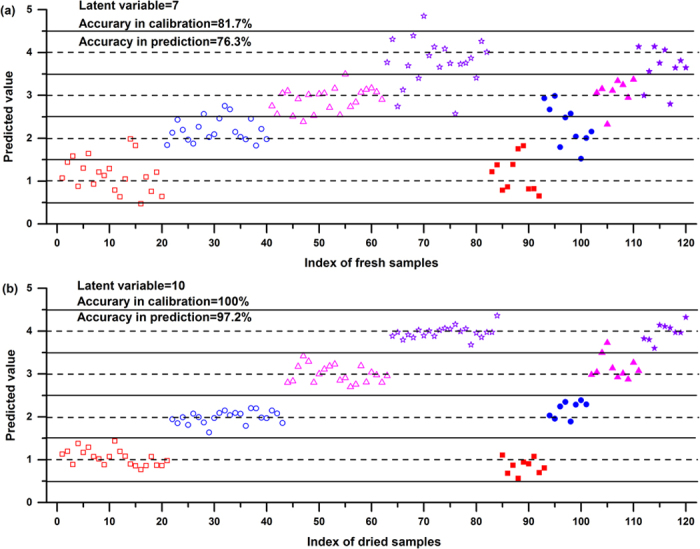
Y-predicted plot for PLS-DA classification of different symptoms of infected plants based on full spectrum of (**a**) fresh samples and (**b**) dried samples. Red squares, blue circles, magenta up triangles, violet stars indicate healthy, mild-infected, moderate-infected, severe-infected tobacco samples, respectively. Hollow markers indicate calibration set while solid markers indicate prediction set. The classification performance based on dried samples were higher than that based on fresh samples, with the accuracy of 97.2% in the prediction set.

Symptom discrimination results by PLS-DA are provided in Fig. 4. It was noted that both fresh and dried samples achieved acceptable results, and the result of model based on dried samples performed better than that of fresh samples. In detail, we obtained the average accuracy for dried samples of 100% in the calibration set, and 97.2% in the prediction set. Only one moderate-infected sample was misclassified as severe-infected sample in the prediction set. The results obtained in PLS-DA models were in accordance with PCA models, which indicated the existence of moisture content in sample might worsen the performance of disease discrimination. Although little sample preparation was required when detecting using fresh samples, the classification performance should be further improved.

**Figure 5:**
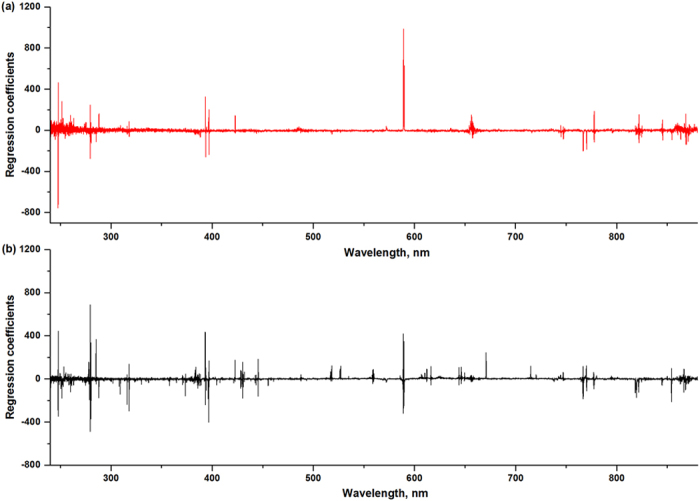
Regression coefficients for PLS models based on (**a**) fresh samples and (**b**) dried samples. A variable with large absolute value of regression coefficient plays an important role in PLS regression. The variables that actually worked in PLS models were main observed emission lines.

In addition, we calculated the regression coefficients of PLS (shown in Fig. 5), which could examine the contribution of each variable in PLS model. A variable with large regression coefficient played an important role in PLS regression. In other words, large positive value meant a positive link with the symptom of TMV, and negative value showed a negative link, while the variable with little value could be considered as noise or irrelative information. As shown in Fig. 5, the variables with large regression coefficients corresponded to the main observed emission lines. Since more emission lines were noted from the spectra based on dried samples, there were more variables contributing the PLS regression. In addition, there were several emission lines, such as C I (247.86 nm), Si I (251.61 nm), Mg I (279.55 nm), Ca II (393.37 nm, 396.85 nm), Na (589.00 nm, 589.59 nm), etc., which played important roles in both fresh and dried samples. The variance of these elements might relate to the degrees of symptoms of infected plants.

### Classification based on observed emission lines

As mentioned before, LIBS spectra usually contain thousands of variables, and lots of them are noise or irrelative signal for disease detection. In order to eliminate the irrelative signal, as well as increasing the calculation speed, we wanted to search for the discriminating emission lines. Since the variables that actually worked in PLS models were mainly observed emission lines, in this case, we further established PLS models using the observed emission lines. In addition, SVM models were also established to compare the modeling capability of nonlinear and linear algorithm. In total, 93 emission lines for dried pellets and 56 emission lines for fresh leaves were used for modeling. The number of samples used for calibration, cross-validation and prediction in the classification based on observed emission lines was as the same as the number used in full spectrum classification. Detailed information of these emission lines has been stated in Table 1.

As shown in Table 3, the PLS classification based on observed emissions obtained acceptable results. However, the classification accuracy was reduced in some extent, which was likely due to the loss of some useful information. This useful information could be the profile of the peaks or some little peaks ignored in this case. Similar to the previous results when all the variables of the spectra were involved, PLS model based on the spectra of dried samples obtained better classification than that based on fresh samples. The Y-predicted values for the dried samples were much closer to the ideal values of group labels. In detail, the classification accuracy for dried samples was 91.7% in the calibration set and 88.9% in the prediction set, while for fresh samples the classification accuracy was 67.1% and 63.2%, respectively.

In addition, the results of SVM models outperformed those of PLS-DA models (see Table 3). The models of both fresh and dried samples had good classification results, achieving the accuracy in the prediction set higher than 90%. In fresh samples, only two samples were misclassified, one in mild-infected samples was misclassified as severe-infected samples, and one in severe-infected samples was misclassified as moderate-infected sample. In dried samples, two in severe-infected samples were misclassified as moderate-infected samples. Besides, all healthy tobaccos were correctly classified. It was mainly credited to the capability of SVM to deal with nonlinear classification case, which has also been proven by Cisewski *et al*. and Dingari *et al*.^16^,^17^. The nonlinear relationship in this case might be due to the ‘matrix effect’ and complex ablation process. Besides, the classification accuracy in the cross-validation set decreased in both PLS-DA and SVM models when using the spectral data from fresh leaves. It indicated that moisture content worsened the classification performance in both linear and nonlinear model, while the influences might be alleviated in nonlinear model.

## Discussion

The results demonstrated that LIBS combined with chemometrics could provide a novel approach to detect TMV-infected tobacco. Compared with other detection methods, this approach had the advantages of fast analysis speed, simple sample preparation and low cost. By ablating the sample simply with a laser, the elemental information represented features of sample could be acquired. Since the infected tobacco might show elemental variations in leaves, this approach provided an opportunity to characterize the features of symptoms, and classify the symptoms.

Herein, we explored the effect of moisture content in samples using different sample preparations (fresh leaves and died leaf pellets). It was proved that the existence of moisture content would worsen the repeatability of LIBS analysis and reduce important features in ‘fingerprint’. In addition, the evaporation of water might be the origin of large value of temperature and electron density observed in fresh samples. The influences of moisture content could also be reflected in classification models, the accuracy of which were all reduced.

For full spectrum, good classification accuracy 97.2% for dried samples and 76.3% for fresh samples were obtained by PLS-DA method. The full spectrum contained thousands of variables, most of which were noise or irrelevant information^18^. As shown in regression coefficient plot, the variables that actually worked in PLS-DA were the main emission lines. As for the observed emission lines, we obtained acceptable results for both fresh and dried samples. However, the classification accuracy was reduced in some extent compared with full spectrum. In addition, we found that nonlinear method (i.e. SVM) could alleviate the influences of moisture, and improve classification accuracy. Using SVM method, the accuracy in cross-validation for fresh samples was increased from 62.2% to 87.8%, and it was increased from 82.1% to 97.6% for dried samples. Actually, the interaction between laser and samples was complex, and it was hard to explain the relationship with linear models^19^. Hence, nonlinear method might be more suitable to this kind of classification, especially when facing complex situations (the influence of ‘matrix effect’ and moisture content were involved).

Although observed emission lines coupled with SVM achieved good classification results for both fresh and dried samples, fresh samples without preparation was preferred for practical application. Because of the high analysis speed and the capability of fieldable application, thousands of fresh leaves could be analysed for preliminary diagnosis and those suspicious samples could be further determined by traditional methods (e.g., PCR). It might reduce the cost in traditional analysis, and also improve the detection accuracy by globe screening.

In addition, the method used here was based on chemometrics, more samples including various symptoms and varieties of plants should be involved in future work. It might help to develop a more robust model, as well as to improve the classification accuracy. Since this experiment was carried out with laboratory instrument, developing fieldable LIBS devices for TMV-infected disease detection had potential and practical meaning for further work. As the development of instruments and analytical methods, LIBS would play an important role in plants disease detection.

## Methods

### Experimental setup

**Figure 6:**
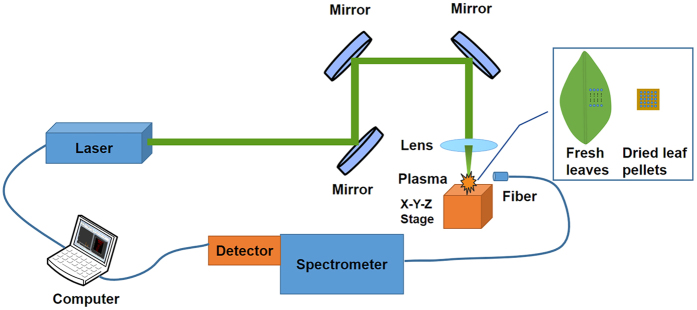
Schematic diagram of experimental LIBS setup. The LIBS setup mainly consists of an Q-switch pulsed laser, optics (mirrors, lens, and fiber, etc.) for guiding the laser pulses onto the samples and transferring the light into a light disperse system, spectrograph for producing the spectra and detection for recoding the signal.

A self-assembled LIBS system was used in this experiment (shown in Fig. 6). A Q-switch pulsed laser (Vlite 200, Beamtech, Beijing, China) was used to ablate samples with the maximal energy of 300 mJ @1064 nm, pulse duration of 8 ns. Since great absorption might occur within fluid medium specimens in the near infrared region, it might be unsuitable to use infrared laser for fresh leaves^20^. In this case, the laser was operated at the second harmonics wavelength with 60 mJ pulse energy and 1 Hz repetition rate. With the help of plano-convex lens (f = 100 mm), the laser beam was focused 2 mm below the sample. Emission spectra were dispersed by an Echelle spectrograph (ME5000, Andor, Belfast, UK) and collected by a ICCD detector (DH334-18F-03, Andor, Belfast, UK). The delay time used in presented experiment was 1.5 μs with the integrated time of 10 μs, and the gain of detector was set at 1500. Before the experiment, wavelength of spectrograph and spectral intensity of detector were calibrated by a mercury argon lamp (HG-1, Ocean optics, USA) and a deuterium tungsten halogen source (DH-2000-BAL-CAL, Ocean optics, USA), respectively.

### Sample preparation

Tobacco seeds MS 87 (provided by Yuxi Zhong Yan Seed Co., Ltd) were used in this experiment. After cultivating on a Murashige and Skoog medium (MS0) containing cell culture vessel for two weeks, the seedlings were transplanted to soil. During the period of vigorous growth, we infected healthy plant with TMV. Mechanical inoculation was used to infect the plants using the method mentioned in Ref. 21. Two in three tobacco plants were inoculated with TMV, and symptom development was monitored daily in the greenhouse. Since the symptom development might vary among different plants, representative tobacco leaves with different symptoms were identified with the help of TMV specialist. For the mild-infected samples, there was a light green coloration between the veins of young leaves, and it was hard to identify with simple visual inspection. For moderate infected samples, the leaves began to appear light-green and dark green area, followed with slight rugosity. For severe-infected samples, the symptom of necrosis appeared in leaves, and the leaves curled severely. In this case, a total of 120 samples with four degrees of symptoms (healthy = 30, mild infected = 30, moderate infected = 30, and severe infected = 30) were collected for LIBS analysis.

For dried leaf pellets, the samples were firstly dried at 80 °C for 4 h in an oven, and grinded by a grinding machine. Then 100 mg of grinded tobacco powders were placed in a square die set, and pressed with 10 tons of pressure for 1 min. Finally, square pellets with side length of 10 mm and thickness of 2 mm were used for analysis. With the help of X-Y-Z stage, ten successive shots each at twenty-five positions were performed for pellets. For fresh leaves, the laser beam ablated one side of the leaves about 5 mm away from the middle of the midribs, and 100 single pulses at different positions were used. In this experiment, the laser beam might pass through fresh leaves, but we didn’t detect extraneous signal from X-Y-Z stage (verified by analysing the spectra when fresh leaves were placed onto a pure copper metal). In order to compare the performance of different sample preparations, the experimental parameters were the same.

### Data analysis

Prior to classification, several data pre-processing procedures were applied for the collected spectra. In order to eliminate the shot-to-shot fluctuation (which may origin from the different ablation efficiency and slight variations of experimental parameters), each spectrum was normalized to the total emission integrated intensity. In addition, a self-developed routine based on median absolute deviation (MAD) method was used to detect outliers^22^. In general, signal variations in LIBS are large compared with other spectral methods (e.g., infrared spectroscopy). It may be credited to the “matrix effect”, and it has been demonstrated that removing the abnormal spectra could help to improve the precision and repeatability of LIBS measurement^23^. MAD is a traditional outlier detection method, and it has the capability to deal with non-normal data in small samples. In this case, we used the peak intensity of emission line CN 388.29 nm as the variable to detect outliers, since CN molecule lines often appear in organic sample and are relatively stable. Then the median and median absolute deviation of the peak intensity (CN 388.29 nm) within spectra from different positions were calculated. A spectrum was considered as outlier when the difference value between its intensity of CN 388.29 nm and median was beyond 2.5 times the MAD. Once the outliers were detected, the spectra were removed from original spectra. We performed this procedure until no outliers were found or the number of remaining spectra was less than 75% of the total number. After outlier detection, all those remaining spectra were averaged to reduce the effect of sample inhomogeneity and the random noise. All the pre-processing operations were performed in Matlab 2014b (The Mathworks Inc., Natick, USA).

In order to investigate the classification of healthy and infected tobacco, PCA was applied to visualize the distribution of symptoms, and PLS-DA and SVM were utilized to quantitatively examine the classification results. PCA^24^ is a traditional multivariate analytical method that reduce the dimensionality of the data by projecting the variables into some principal components with the maximal variations. The main idea of PLS is similar with PCA, which mainly decompresses the independent variables (*X*), and also projects them into new variables (they are called as latent variables in PLS). However, since the dependent variable (*Y*) is also considered in PLS, the loading vectors are related in predicted value^25^. In this experiment, the independent variables were the pre-processed spectra, and the dependent variable was the categorization of each sample (healthy, mild infected, moderate infected, and severe infected). Different from PLS, SVM is a nonlinear algorithm, which is good at dealing with nonlinear classification cases. The basic for SVM is to map the inputs into high-dimensional spaces, and to find a maximum-margin hyperplane that separates the data into two classes^16^. In this study, we used RBF kernel to nonlinearly map samples into higher dimensional space, and two important parameters in RBF kernel (capacity factor, gamma) were determined by performing a grid search. In order to avoid the risk of overfitting, the grid search was performed in a 10-folds cross validation.

All these three chemometrics were performed in the Unscambler X (CAMO AS, Oslo, Norway). Before modelling, original spectra were divided into calibration set and prediction set using k-Means cluster. k-Means cluster is a widely used clustering algorithm, and it was also applied as training set selection method in previous report^26^. Since all samples were categorized as four different groups according to their symptoms, the degree of infection varied slightly even in a single group. Therefore, k-Means can help to pre-cluster in a single categorization, and to improve the classification accuracy. In this case, 11 and 9 clusters were selected for fresh leaves and pellets, respectively. The clustering was performed 100 times to have a robust result.

In addition, LIBS spectra usually contain thousands of variables, while lots of them are usually noise or irrelevant signal for disease detection. Therefore, the classification models based on observed peaks of spectra were also established.

## Additional Information

**How to cite this article:** Peng, J. *et al*. Fast detection of tobacco mosaic virus infected tobacco using laser-induced breakdown spectroscopy. *Sci. Rep.* **7**, 44551; doi: 10.1038/srep44551 (2017).

**Publisher's note:** Springer Nature remains neutral with regard to jurisdictional claims in published maps and institutional affiliations.

## Supplementary Material

Supplementary Figure S1

## Figures and Tables

**Table 1:** Observed emission lines in fresh and dried samples based on NIST database.

Elements	Wavelength (nm)
C (I)	247.86
Si (I)	251.61, 288.16
Fe (I)	293.69*, 385.99*
Fe (II)	253.54*
Mg (I)	277.98*, 285.21, 382.94*, 383.23*, 383.83*, 389.19, 516.73*, 517.27*, 518.36*
Mg (II)	279.08, 279.55, 279.80, 280.27
Ca (I)	422.67, 428.30, 428.94, 429.90, 430.25, 430.77, 431.87, 442.54, 443.57, 457.86*, 458.15*, 458.60*, 487.81, 504.16, 518.88*, 526.22*, 526.56*, 527.03*, 558.20, 558.87, 559.45, 559.85, 560.13*, 585.75*, 610.27, 612.22, 616.22, 616.64*, 643.91*, 644.98*, 646.26*, 647.17*, 649.38*, 671.77*, 714.82*, 720.22*, 854.21
Ca (II)	315.89, 317.93, 370.60, 373.69, 393.37, 396.85, 849.80, 866.21
Mn (II)	292.87*
Sc (II)	364.38*
CN	387.12, 388.29
Al (I)	394.40, 396.15
K (I)	404.41*, 404.72*, 693.88*, 766.49, 769.90
Sr (I)	460.73
Sr (II)	407.77*, 421.55*
Na (I)	589.00, 589.59
Ba (I)	649.88*
H_α_	656.28
Li (I)	670.79*
N (I)	742.36, 744.23, 746.83, 818.49, 821.63, 824.39, 862.92, 868.03
O (I)	777.42, 844.68

Note: the wavelengths that appeared in dried samples while not in fresh samples were marked with star.

**Table 2:** Temperature and electron density of plasma of fresh and dried samples (healthy tobacco leaves).

	Fresh samples	Dried samples
Temperature, K	10807 ± 452	6217 ± 497
Electron density, cm^−3^	(2.74 ± 0.08) × 10^17^	(2.52 ± 0.04) × 10^17^

**Table 3:** Classification results based on observed emission lines.

Methods	Samples	Parameters	Accuracy		
			Calibration	Cross-validation	Prediction
PLS-DA	Fresh leaves	LVs = 7	67.1%	62.2%	63.2%
	Dried leaf pellets	LVs = 6	91.7%	82.1%	88.9%
SVM	Fresh leaves	C = 31.62; G = 0.001778	100%	87.8%	94.7%
	Dried leaf pellets	C = 17.78; G = 0.0004642	100%	97.6%	94.4%

Abbreviations: LVs, latent variables; C, capacity factor; G, gamma.
