# Supplementary material for: Fast detection of tobacco mosaic virus infected tobacco using laser-induced breakdown spectroscopy
Source: Sci Rep. 2017 Mar 16;7:44551. doi: 10.1038/srep44551 (PMC5353609; doi:10.1038/srep44551)

Fast detection of tobacco mosaic virus infected tobacco using laser-induced breakdown spectroscopy

Jiyu Peng[1], Kunlin Song[1], Hongyan Zhu[1], Wenwen Kong[1,2], Fei Liu[1*], Tingting Shen[1] and Yong He[1]

[1]College of Biosystems Engineering and Food Science, Zhejiang University, Hangzhou, 310058, China

[2]School of Information Engineering, Zhejiang A&F University, Lin'an, Hangzhou, 311300, China

*Corresponding author: Fei Liu, Email: fliu@zju.edu.cn, Tel./Fax: +86-571-88982825

Supplementary Figure S1:

Loadings of the first three PCs for (a) fresh leaves and (b) dried pellets. The variables from main emission lines projected to the three PCs, which played an important role in classification. The variables with large loadings corresponded to the main emission lines, and more variables with large loadings were observed in dried pellets.

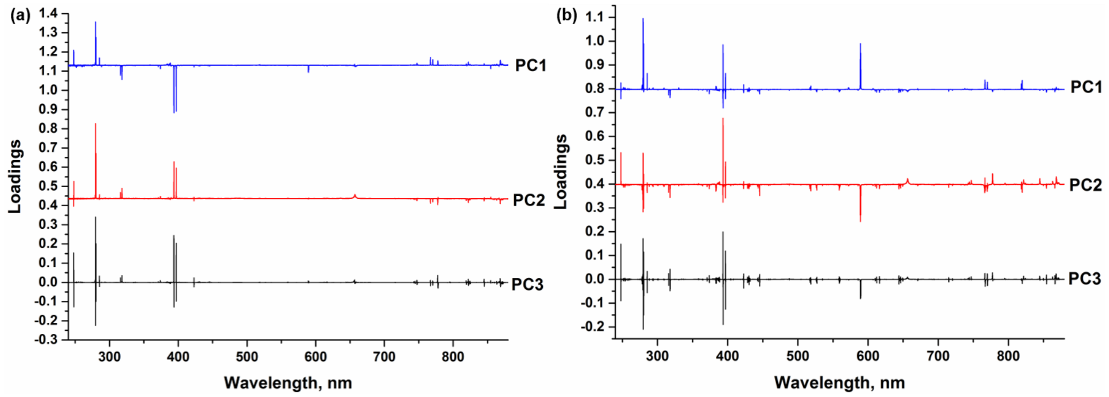

Supplement: Supplementary Figure S1 [file srep44551-s1.pdf]
